# Characterization of the epidermal-dermal junction in hiPSC-derived skin organoids

**DOI:** 10.1016/j.stemcr.2022.04.008

**Published:** 2022-05-12

**Authors:** Veronika Ramovs, Hans Janssen, Ignacia Fuentes, Amandine Pitaval, Walid Rachidi, Susana M. Chuva de Sousa Lopes, Christian Freund, Xavier Gidrol, Christine L. Mummery, Karine Raymond

**Affiliations:** 1 Department of Anatomy and Embryology, Leiden University Medical Center, Leiden, the Netherlands; 2 Electron Microscopy Facility, Netherlands Cancer Institute, Amsterdam, the Netherlands; 3 Fundación DEBRA Chile, Santiago, Chile; 4 Centro de Genética y Genómica, Facultad de Medicina, Clínica Alemana Universidad del Desarrollo, Santiago, Chile; 5 LUMC hiPSC Hotel, Leiden University Medical Center, Leiden, the Netherlands; 6 University of Grenoble Alpes, CEA, INSERM, IRIG-BIOMICS, Grenoble, France; 7 Ghent Fertility and Stem Cell Team (G-FaST), Department for Reproductive Medicine, Ghent University Hospital, Ghent, Belgium

**Keywords:** hiPSC-derived hair-bearing skin organoids, epidermal-dermal junction, collagen

## Abstract

Human induced pluripotent stem cell (hiPSC)-derived hair-bearing skin organoids offer exciting new possibilities for modeling diseases like epidermolysis bullosa (EB). These inherited diseases affect 1 in 30,000 people worldwide and result from perturbed expression and/or structure of components of the epidermal-dermal junction (EDJ). To establish whether hiPSC-derived skin organoids might be able to capture salient features of EB, it is thus important to characterize their EDJ. Here, we report successful generation of hair-bearing skin organoids from two hiPSC lines that exhibited fully stratified interfollicular epidermis. Using immunofluorescence and electron microscopy, we showed that basal keratinocytes in organoids adhere to laminin-332 and type IV collagen-rich basement membrane via type I hemidesmosomes and integrin β1-based adhesion complexes. Importantly, we demonstrated that EDJs in organoids are almost devoid of type VII collagen, a fibril that mediates anchorage of the epidermis to dermis. This should be considered when using skin organoids for EB modeling.

## Highlights

• Hair-bearing skin organoids form a stratified interfollicular epidermis *in vitro*
• Organoid keratinocytes form type I hemidesmosomes and integrin β1-based complexes
• Basement membrane in skin organoids is rich in laminin-332 and type IV collagen
• Low levels of type VII collagen in organoids may be limiting for disease modeling

## Teaser

In this article, Raymond and colleagues present the generation of hair-bearing skin organoids from two human induced pluripotent stem cell lines. Results include a thorough characterization of the epidermal-dermal junction and highlight that skin organoids only express low levels of type VII collagen that may limit their use for disease modeling, notably epidermolysis bullosa.

## Introduction

Skin integrity depends on the epidermal-dermal junction (EDJ), the interface between basal keratinocytes of the epidermis and stroma of the dermis. Basal keratinocytes adhere to the underlying basement membrane (BM), an extracellular matrix (ECM) sheet, composed primarily of laminin and type IV collagen (ColIV) networks (Burgeson and Christiano, 1997). This adhesion is mediated by integrins, transmembrane αβ heterodimer proteins that cluster to adhesion structures on the basal plasma membrane of keratinocytes. In physiological conditions, keratinocytes highly express collagen-binding integrin α2β1, laminin-332/-511-binding α3β1, and laminin-332-binding α6β4. The adhesive integrity of the EDJ largely depends on α6β4-containing adhesion structures called hemidesmosomes (HDs). In contrast to simple epithelia (e.g., intestine), which express type II HDs consisting solely of the integrin α6β4 and plectin, keratinocytes in the EDJ adhere via type I HDs that additionally contain tetraspanin CD151 and bullous pemphigoid antigens 180 (BP180) and 230 (BP230) (Margadant et al., 2010). Additionally, BM is connected to the dermal connective tissue via anchoring fibrils, consisting of ColVII (Burgeson and Christiano, 1997).

Aside from its function in epidermal adhesion, the EDJ also regulates the permeability barrier of the skin and plays a role in epithelial-mesenchymal interactions and signal transduction. Its pivotal role in skin physiology is reflected in severe pathologies that occur when the function of EDJ components is impaired. This is the case in, for example, epidermolysis bullosa (EB), which is caused by, among others, mutations in genes encoding components of HDs and associated filaments. These include mutations in the *COL7A1* gene (coding for ColVII) causing dystrophic EB, a severe blistering disease (Turcan and Jonkman, 2015). Furthermore, EDJs, particularly BMs and integrins, play an important role in initiation and progression of skin cancers (Chang and Chaudhuri, 2019; Ramovs et al., 2016).

The generation of hair-bearing skin organoids derived from human pluripotent stem cells (hPSCs) has recently been described (Lee et al., 2020). These clearly offer exciting new opportunities for skin disease modeling, regenerative medicine, and developmental research. However, to grasp the full potential of this model, it is essential to understand the structure and developmental stage of EDJs formed in the skin organoids. This is especially important since their gene expression profile and hair morphology indicate that they represent mid-gestational skin, a fetal stage during which the main components of the EDJ gradually begin to develop (Nicholas and Jacques, 2005). In addition, inherent differences between hPSC lines can lead to variable outcomes (Lee and Koehler, 2021) and therefore require the validation of protocols with independent cell lines.

In this study, we report the generation of hair-bearing skin organoids from two male human induced pluripotent stem cell (hiPSC) lines and address the organization of their EDJ using immunofluorescence (IF) and transmission electron microscopy (TEM).

## Results and discussion

### Successful generation of hair-bearing skin organoids from two hiPSC lines

**Figure 1:**
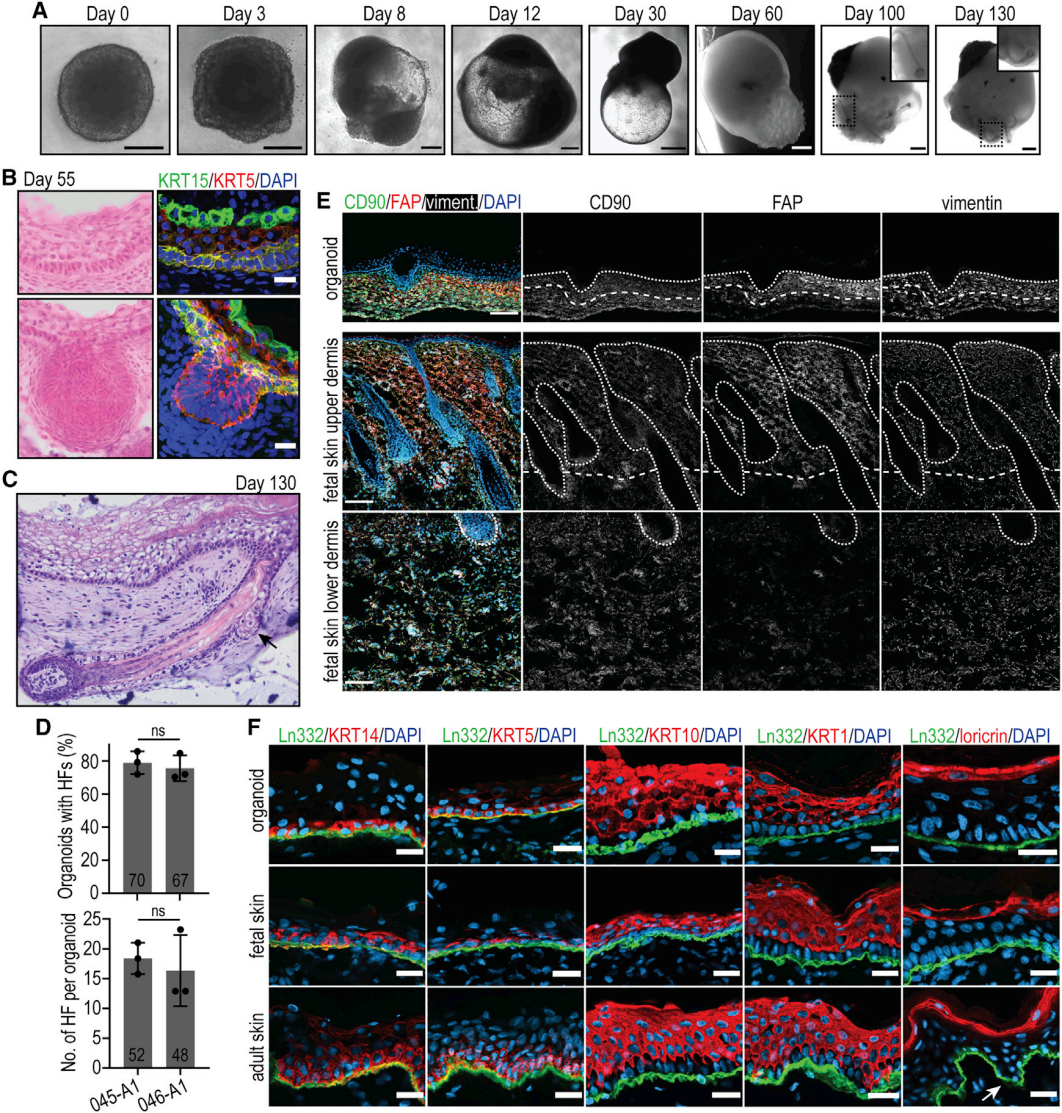
HFs, dermal fibroblasts, and stratification of interfollicular epidermis in hiPSC-derived skin organoids (A) Representative bright-field images of the organoids at different stages of development. Scale bars: 200 μm (days 0–12) and 400 μm (days 30–130). (B) Representative bright-field (left) and confocal (right) images of organoid sections at day 55, stained with H&E (left) or with anti-KTRT5 and KRT15 antibodies (right). Scale bar: 20 μm. (C) Representative bright-field image of H&E-stained section of skin organoid at day 130 showing stratified epidermis and fully developed HFs, equipped with sebaceous gland (arrow). (D) Quantification of the percentage of organoids with HFs (left) and the number of HFs per organoid (right) derived from LUMCi045-A1 (045-A1) and LUMCi046-A1 (046-A1) lines at day 130. The mean ± SD of 48-70 organoids from 3 independent differentiations is shown. (E) Characterization of dermal fibroblasts in organoid (day 130) and fetal human skin sections. Vimentin^+^ fibroblasts cluster with the FAP^+^ population in the upper dermis and the FAP^low^ CD90^+^ population in the lower dermis (delineated with dashed line). Upper dashed line: BM. Scale bar: 100 μm. (F) Confocal images of organoid (day 130) and adult and fetal human skin sections stained with anti-laminin-332 antibody as well as with anti-KTRT14, KRT5, KRT10, KRT1, or loricrin antibodies. Note that the stratification of the skin organoids (top) is comparable to that of human epidermis (bottom). Arrow: rete ridges, present only in adult human skin. Scale bars: 20 μm.

Using two independent control hiPSC lines (LUMCi045-A1 and LUMCi046-A1), we were able to recapitulate the *in vitro* skin organogenesis described by Lee et al. (2020) (Figure 1A). Consistent with the original protocol, the majority of skin organoids became bipolar at around day 30 of directed differentiation, with an opaque cell mass (“tail”) at the pole opposite to the translucent epidermal cyst (“head”) (Figure 1A). At around day 50, the epithelium was composed of a basal layer of keratin 5 (KRT5)^+^ keratin 15 (KRT15)^+^ cells, an intermediate KRT5^low^ layer, and a KRT5^low^ KRT15^high^ periderm-like layer (Figure 1B). Hair placodes were observed at around day 60, and fully grown hair follicles (HFs) developed after 100 days in culture (Figures 1A–1C). At day 130, 78.9% and 75.7% of the skin organoids derived from LUMCi045-A1 and from LUMCi046-A1 displayed HFs, with a mean number of 18 and 16 HFs per organoid, respectively (Figure 1D). HFs showed similar organization to that previously reported in organoid-derived HFs, occasionally containing sebum-producing sebaceous glands (Figures 1C, S1A, and S1B) and innervation as illustrated by TUJ1^+^ neuronal processes and TUJ1^+^ Merkel-like cells (Figure S1C) (Lee et al., 2020). When pigmented, the matrix region of the HFs and interfollicular epidermis were enriched with PMEL^+^ melanocytes (Figures S1D and S1E). Skin organoids were rich in vimentin^+^ dermal fibroblasts, which, much like mid-gestational fetal skin, clustered to FAP^high^ papillary fibroblasts in the upper dermis and CD90^high^ FAP^low^ reticular fibroblasts in the lower dermis (Figures 1E and S1D) (Korosec et al., 2019). Consistent with the previous report, we also observed hyaline cartilage in all organoids analyzed (Figure S1F) (Lee et al., 2020).

### hiPSC-derived skin organoids form a stratified interfollicular epidermis

At day 130, skin organoids formed a stratified epidermis, which resembles the stratification of the adult human skin: basal keratinocytes express KRT5 and KRT14, and suprabasal keratinocyte layers contain KRT1 and KRT10, with a terminally differentiated loricrin-positive cornified layer marking the upper end of the epidermis (Figures 1C and 1F). Such complete stratification of organoid interfollicular epidermis was shown previously when mature organoids were grafted in a mouse model (Lee et al., 2020), but our data support the notion that organoid grafting may not be necessary for short-term experiments that require mature stratification. Nonetheless, it is important to note that organoid grafting in mice leads to the formation of rete-ridge-like structures, which are present in human skin but which neither we nor Lee et al. (2020) observed in the epidermis of skin organoids (Figure 1F). As rete ridges develop after week 19 of gestation (Figure 1F), this likely indicates increased maturity of the model after grafting. Furthermore, mechanical stress has been shown to play an important role in the development of rete ridges (Penrose and Ohara, 1973; Xiong et al., 2013), thus their absence might also reflect the lack of mechanical stress in the *in vitro* culture.

### BM underlies the epidermis of hiPSC-derived skin organoids

**Figure 2:**
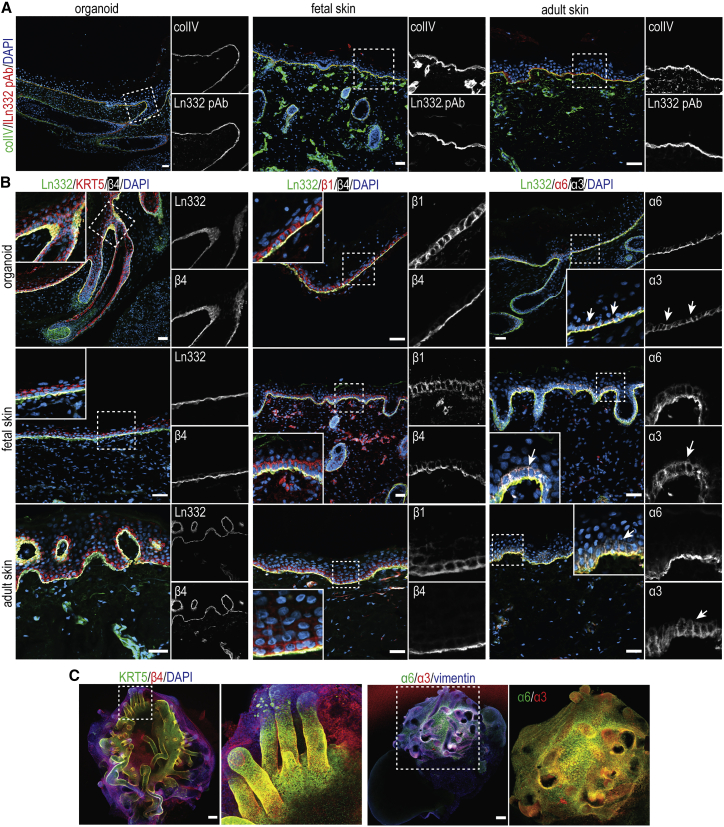
Characterization of the basement membrane of hiPSC-derived skin organoids (A) Representative confocal images of organoid and human adult and fetal skin sections stained with anti-collagen IV and anti-laminin-332 antibodies. Scale bars: 40 μm. (B) Representative confocal images of organoid and human skin sections stained for laminin-332 in combination with keratin 5 (KRT5) and integrin β4 subunit (left panels), integrin β1 and integrin β4 subunits (middle panels), or integrin α6 and integrin α3 subunits (right panels). The distribution of laminin-332 and integrin subunits α3, α6, β4, and β1 in skin organoids is similar to that in adult and mid-gestational fetal human skin. Arrows point to the localization of integrin α3β1 to cell-cell contacts. Scale bars: 40 μm. (C) Confocal images of organoid whole mounts stained with anti-KRT5 and anti-β4 integrin antibodies (left panels) or with anti-vimentin and anti-α3 and -α6 integrin subunit antibodies (right panels). Note the homogeneous distribution of integrins α3β1 and α6β4 to the polarized head of organoids. Scale bars: 200 μm. Organoids are imaged at day 130. Dashed boxes are magnified. Confocal images are presented as maximum projected z stacks.

Basal keratinocytes adhere to the BM, an ECM sheet composed of ColIV and laminin networks, which can be detected as early as week 7 of fetal development (Hertle et al., 1991). Epidermal BM predominantly contains laminins-332 and -511, of which laminin-332 functions as a main anchoring filament for basal keratinocytes (Burgeson and Christiano, 1997; Pozzi et al., 2017). In organoids, both laminin-332 and ColIV showed homogeneous expression, restricted to the BM (Figures 1F and 2A). Consistent with their role in adhesion to the BM, all subunits of the major epidermal integrins could be detected at the EDJ in the epidermal cyst of organoids: α3, α6, β4, and β1 (Figures 2B and 2C). In adult skin, integrin α6β4 mainly localizes to the basal membrane of basal keratinocytes, whereas its expression is pancellular early during fetal development, with progressive restriction to the basal membrane from week 10 of gestation (Hertle et al., 1991). The stratified epidermis of immature organoids (day 55) resembles early stages of skin development with a strong expression of integrin α6β4 also at the lateral and apical membranes of basal keratinocytes (Figure S2); this becomes more restricted to the basal membrane in mature organoids (day 130), reflecting the organization of human skin from mid-gestation onward (Figure 2B). Integrin α3β1 can be observed at basal and lateral membrane of keratinocytes at EDJ of organoids, which is in line with its localization in mature epidermis (Figure 2B, white arrows) (Zuidema et al., 2020).

**Figure 3:**
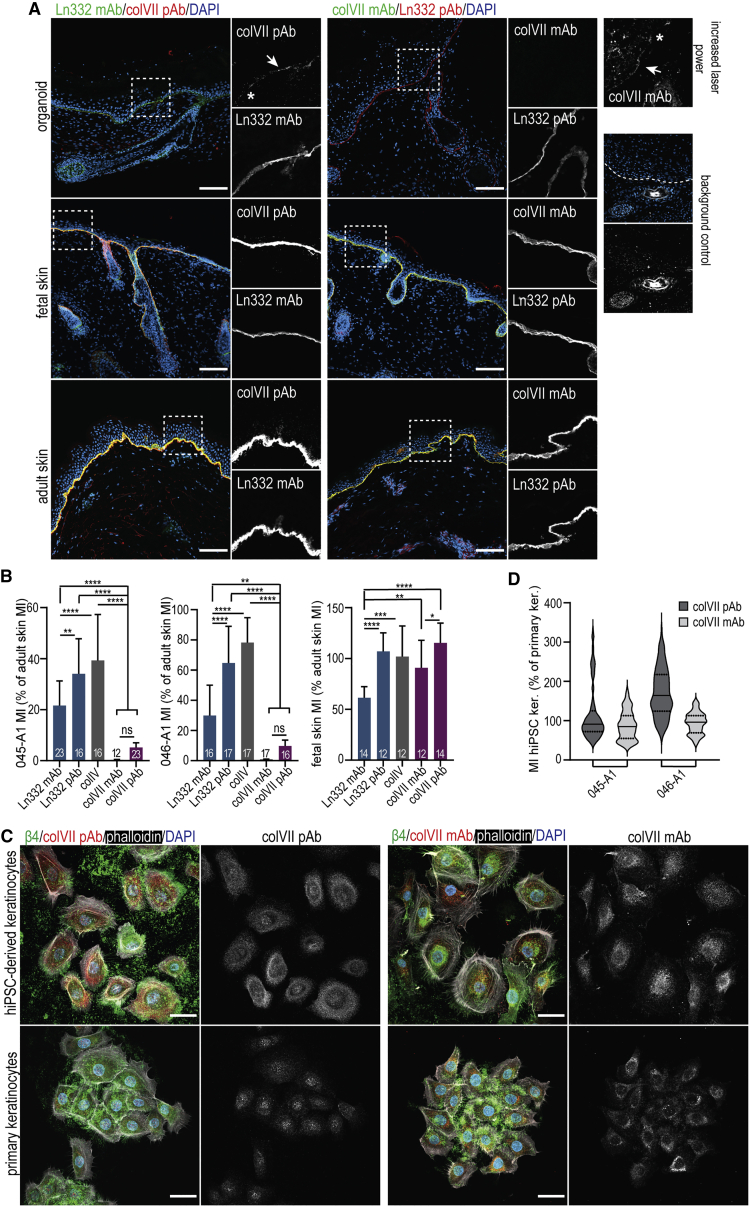
Expression levels of ColVII in skin organoids (A) Representative confocal images of organoid (day 130) and human fetal and adult skin sections stained with anti-laminin-332 and anti-ColVII antibodies. The expression of ColVII is low in the skin organoids and often non-uniform (arrows), with some areas seemingly devoid of any ColVII fibers (asterisk). The panels “background control” represent the signal observed when using secondary antibodies only, with a similar setting to those applied for the condition “increased laser power.” Dashed line marks the BM. Dashed boxes are magnified. Confocal images are presented as maximum projected z stacks. Scale bar: 100 μm (B) Quantification of the laminin-332, ColIV, and ColVII mean intensity (MI) in skin organoids (day 130) derived from LUMCi045-A1 (left), LUMCi046-A1 (middle) lines, and fetal skin (right) as compared with adult human skin. A total of 5–6 images of 2–4 independent differentiations of hiPSC or of human samples was used for quantifications (Tukey’s multiple comparison, n = 12–23, ^∗∗^p < 0.01, ^∗∗∗∗^p < 0.0001, mean ± SD). (C) Representative images of primary and hiPSC-derived keratinocytes stained with anti-ColVII and integrin β4 antibodies and phalloidin. Scale bars: 30 μm. (D) Violin plots showing the ColVII expression of hiPSC-derived keratinocytes as a percentage of ColVII MI of primary keratinocytes. Line: median; dashed lines: quartiles; n = 142–223 β4-positive cells.

Next, we examined the expression of ColVII, which anchors BM to the dermis, using polyclonal and monoclonal antibodies. While homogeneous and abundant in adult skin, ColVII expression was rather heterogeneous and barely detectable along the EDJ of skin organoids. By contrast, all other ECM components of the EDJ that were examined were clearly detectable, albeit at reduced levels compared with human skin (Figures 3A, 3B, and S3A). In line with this, the expression of *COL7A1* was lower in organoids compared with adult human skin when normalized to the expression of two chains encoding laminin-332 and two chains encoding ColIV (Figure S3B).

The low levels of ColVII are remarkable considering that weak expression of this protein has been detected as early as at 8 weeks of gestation in fetal skin and that homogeneous interfollicular expression has been observed from 13 weeks of gestation onward (Karelina et al., 2000; Ryynänen et al., 1992). Indeed, analysis of 19-week-old fetal skin revealed comparable levels of ColVII to those in adult human skin (Figures 3A and 3B). Given that skin organoids resemble fetal skin of gestational week 18 (Lee et al., 2020), low and heterogeneous levels of expression of ColVII were not expected. To rule out any specific defects in the hiPSC lines used in this study, we quantified the expression of ECM components in organoids generated from previously published female hiPSC line WT2. The levels of ColVII in WT2 organoids were similar to those observed in LUMCi045-A1 and LUMCi046-A1 (Figures S3C and S3D). We further differentiated LUMCi045-A1 and LUMCi046-A1 directly to keratinocytes and compared their levels of ColVII with those in primary human keratinocytes. When grown on glass coverslips, hiPSC-derived and primary keratinocytes produced comparable amounts of ColVII (Figures 3C, 3D, and S3E–S3I), suggesting that low levels of ColVII observed in organoids are model dependent. Remarkably, skin organoids did not show obvious morphological defects in the EDJ area, which would be expected in light of the weak anchoring of the epidermis. This could be explained by the lack of exposure to mechanical stress; furthermore, low mechanical stress could by itself affect the assembly of collagen fibrils (Kubow et al., 2015). It would be interesting to determine whether aberrant expression of ColVII could be resolved by maturation and/or changes in mechanical stress; analysis of grafted organoids could be informative in this regard. Importantly, the aberrant levels of ColVII could be a limiting factor for the use of skin organoids for *in vitro* disease modeling: its low expression does not only result in severe types of EB, but ColVII also plays an important role in wound healing and epidermal squamous cell carcinoma (Chung and Uitto, 2010; Nyström et al., 2013).

### Basal keratinocytes in skin organoids adhere to the BM via type I HDs and integrin β1-based adhesion complexes

**Figure 4:**
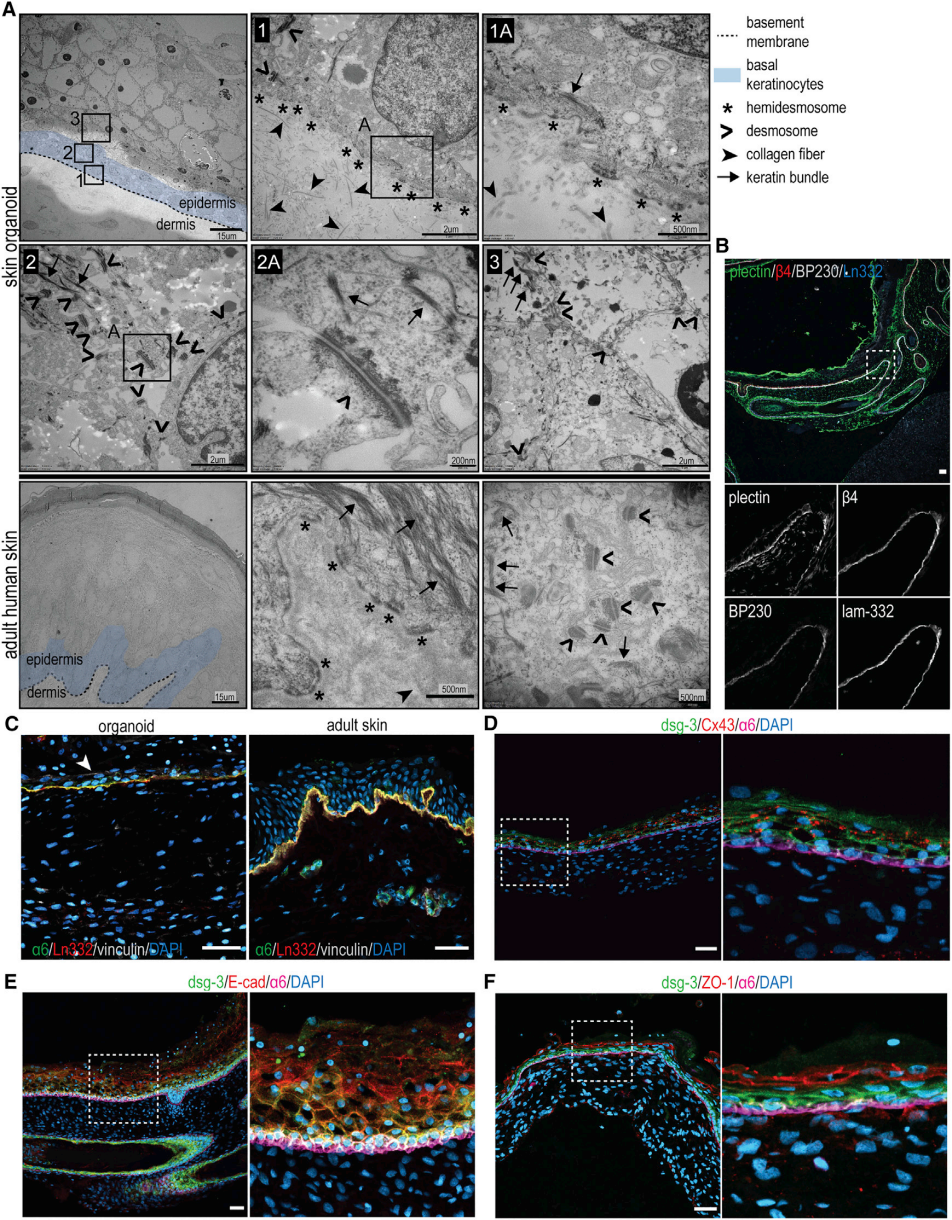
Basal keratinocytes adhere to the basement membrane via type I hemidesmosomes (A) Representative TEM images of the organoid and adult human epidermis. HDs (asterisk) are observed at the basal membrane of the basal keratinocytes (blue), adhering to the underlying basement membrane (dashed line). Desmosomes can be detected in cell-cell adhesions sites in different layers of the stratified epidermis. (B) Representative confocal images of skin organoid showing colocalization of plectin, β4 integrin, BP230, and laminin-332. This identifies HDs in organoids as type I, which are typically found in the adult human skin. (C) Representative confocal images of organoid and human skin sections stained with anti-α6 integrin, anti-laminin-332, and anti-vinculin antibodies. Whereas vinculin is restricted to the BM in the adult skin, its expression can be observed also at the lateral and apical membranes of basal and suprabasal keratinocytes in skin organoids (arrowhead), which is similar to that observed during the mid-gestation period of human fetal development (Figure S4). (D–F) Representative confocal images of skin organoids stained for desmosomal marker-desmoglein-3 (dsg-3) and integrin α6 together with (D) gap junction marker connexin 43 (Cx43), (E) adherens junction marker E-cadherin (E-cad), or (F) tight junction marker ZO-1. Note the lower expression of dsg-3 in the upper layers of epidermis. Dsg-3, Cx43, E-cad, and ZO-1 localize to the cell-cell junctions. Dashed boxes are magnified to the right. (B–E) Scale bars: 40 μm. Confocal images are presented as maximum projected z stacks. All organoids are imaged at day 130.

Firm adhesion of keratinocytes to the BM is mediated by HDs, which link the keratin cytoskeleton to the laminin-332 via the integrin α6β4. Since impaired function of virtually any HD component leads to the development of EB, we thought it was imperative to investigate to what extent skin organoids form HDs (Margadant et al., 2010). Using TEM, we detected numerous HDs localized at the basal membrane of the keratinocytes in EDJ (Figure 4A). Next, we investigated the expression of BP230, a plakin that, in addition to plectin, connects integrin α6β4 to keratin cytoskeleton in more complex type I HDs, which are normally found in the epidermis (Margadant et al., 2010). We observed colocalization of BP230 with HD components α6β4, plectin, and laminin-332, confirming the presence of type I HDs in organoids (Figure 4B).

Integrins consisting of β1 subunits, particularly α3β1 and α2β1, also contribute to the adhesion of basal keratinocytes by linking the ECM to the actin cytoskeleton via adaptor proteins talin and vinculin (Margadant et al., 2010). Whereas vinculin is restricted to the BM in adult skin, its expression can also be observed at the lateral and apical membranes of basal and suprabasal keratinocytes in skin organoids (Figure 4C); this resembles vinculin localization in skin at mid-gestation of human fetal development (Figure S4) (Hentula et al., 2001). Integrin α3β1- and α2β1-containing adhesion structures also play a role in the migration and spreading of keratinocytes during non-homeostatic conditions, such as wound healing, as well as in maintaining cell-cell contact integrity (Zuidema et al., 2020). Keratinocytes in epidermis form cell-cell adhesions via E-cadherin (E-cad)^+^ adherens junctions, desmoglein-3 (dsg-3)^+^ desmosomes, zonula occludens-1 (ZO-1)^+^ tight junctions, and connexin 43 (Cx43)^+^ gap junctions (Churko and Laird, 2013; Sumigray and Lechler, 2015). All main adhesion junctions were observed in skin organoids at expected sites (Figures 4A and 4D–4F), further confirming the relatively mature state of skin stratification.

In summary, we were able to generate hair-bearing skin organoids from two independent hiPSC lines following the multistep protocol recently developed by Lee et al. (2020). Moreover, we thoroughly characterized their EDJ. We showed that skin organoids form a fully stratified interfollicular epidermis *in vitro.* Basal keratinocytes in organoids adhere to laminin-332 and ColIV-rich BM via type I HDs and integrin β1-based adhesion complexes. The EDJ in organoids derived from our cell lines was almost devoid of ColVII, indicating that further maturation is required to take full advantage of skin organoids as disease model for some forms of EBs, in particular those caused by mutations in the *COL7A1* gene (Table S1).

## Experimental procedures

### Cell culture

LUMCi045-A1, LUMCi046-A1, and WT2 hiPSC lines were previously described (Ramovs et al., 2021; Zhang et al., 2014). They were maintained in StemFlex medium (Thermo Fisher Scientific, #A3349401) on vitronectin-coated plates (Stemcell Technologies, #07180) and passaged twice a week as single cells by using Gentle Cell Dissociation Reagent (Stemcell Technologies, #07174) at a density of 2.5 × 10^4^ cells/cm^2^. Keratinocytes were differentiated from LUMCi045-A1 and LUMCi046-A1 as described previously and cultured in CnT-07 (Bio-Connect) (Guo et al., 2013, supplemental experimental procedures). Primary human keratinocytes were isolated from breast skin tissue explants as previously described (Auxenfans et al., 2012) and cultured in serum-free keratinocyte medium (Thermo Fisher Scientific, #17005042) supplemented with 50 μg/mL bovine pituitary gland extract, 2.5 ng/mL epidermal growth factor, and antibiotics. Donors signed informed consents, and the study was approved by the French research ministry (DC-2020-4338). HaCaT keratinocytes were cultured in CnT-07. All cell lines were kept at 37°C, 5% CO2, and 20% O2.

### Generation of skin organoids and quantification of HF-formation frequencies

Hair-bearing skin organoids were generated as previously described (Lee and Koehler, 2020). Skin organoids that did not show bipolar organization at around day 30 were excluded from the study. Quantifications of the percentage of organoids producing HFs and of the number of HFs per skin organoid were performed at day 130 in 3 independent differentiations for both hiPSC lines, respectively. Images of each organoid were taken with a Leica M420 macroscope using an Olympus XC50 digital color camera. The angle of the organoids revealing the highest number of HFs was selected for quantification.

### Human tissue specimens

Adult human skin samples used in this study were obtained upon mammoplasty or abdominoplasty of females of 32- to 40-years-old with type I/II skin, based on the Fitzpatrick skin-type classification scale. The human fetal tissue used in this work (19 weeks, facial area) was obtained from elective abortion (without medical indication) with signed informed consent from all donors. The work described here was reviewed and approved by the Medical Ethical Committee of Leiden University Medical Center (P08.087).

### Antibodies

Primary antibodies used are listed in Table S2, and secondary antibodies are listed in supplemental experimental procedures.

### IF microscopy

For cryosections, organoids were embedded in 15% sucrose/7.5% gelatin solution as previously described (Bajanca et al., 2004). Sections (10 μm thick) were fixed for 10 min in ice-cold acetone (or 15 min in 4% paraformaldehyde when stained with Nile Red) and blocked with 2% bovine serum albumin (BSA; Sigma) in PBS for 1 h at room temperature (RT). hiPSC-derived and primary keratinocytes were seeded on coverslips and fixed after 4 days with 2% paraformaldehyde for 30 min, permeabilized with 0.2% TritonX-100 for 5 min, and blocked with PBS 2% BSA for 1 h at RT. Incubations with primary and secondary antibodies were performed in PBS 2% BSA for 1 h at RT. For Nile Red staining, sections were further incubated for 15 min in Nile Red/PBS (0.05 μg/mL). Nuclei were stained with DAPI. When indicated, filamentous actin was visualized using Alexa Fluor 647-conjugated phalloidin (Thermo Fisher Scientific, #A22287). Sections were mounted in Fluoromount-G® (SouthernBiotech) and coverslips in Mowiol. For whole-mount immunostaining, organoids were processed as previously described (Lee et al., 2020). Cryosections (human skin and day-130 organoids) and keratinocytes were analyzed with a Zeiss LSM900 Airyscan2 upright confocal microscope, and cryosections of day-55 organoids as well as whole mounts were analyzed with a Leica TCS SP5 confocal microscope. Images of maximum intensity projection are presented. Images of skin organoids developed from the LUMCi045-A1 hiPSC line are shown. All images were processed using Fiji/ImageJ (Rueden et al., 2017; Schindelin et al., 2012). Detailed explanation of the quantification of the ECM components can be found in supplemental experimental procedures.

### Histology

Cryosections of skin organoids were stained for hematoxylin and eosin (H&E). Images were taken with an Olympus AX70 microscope using an Olympus XC50 digital color camera.

### TEM

For TEM, tissue samples were fixed in Karnovsky’s fixative. Post-fixation was done with 1% osmiumtetroxide in 0.1 M cacodylatebuffer. Organoids were washed and stained *en bloc* with Ultrastain 1 (Leica, Vienna, Austria), followed by ethanol dehydration series. The samples were embedded in a mixture of DDSA/NMA/Embed-812 (EMS, Hatfield, PA, USA), sectioned, and analyzed with a Tecnai12G2 electron microscope (Thermo Fischer Scientific, Eindhoven, the Netherlands). Images of skin organoids developed from the LUMCi045-A1 hiPSC line are shown.

### Statistical analysis

Statistical analysis was performed using GraphPad Prism (v.9.1.1). Unpaired two-tailed t test was used to compare two experimental groups. Experiments with more than two experimental groups were analyzed using one-way ANOVA. Comparisons were conducted using Tukey’s multiple comparison test after a global ANOVA was determined to be significant. Results with p values lower than 0.05 were considered significantly different from the null hypothesis.

## Author contributions

K.R. and V.R. designed and performed the experiments and wrote the manuscript. H.J. performed TEM, I.F. provided samples for the generation of hiPSC lines, A.P. and W.R. provided primary keratinocytes, and S.M.C.d.S.L. collected and isolated the fetal human skin. K.R., C.F., X.G., and C.L.M. provided supervision and discussion. All authors read and approved the manuscript.

## Conflicts of interest

C.L.M. is associate editor of *Stem Cell Reports*.

## Supplemental information

Document S1. Figures S1–S4, Tables S1–S3, and Supplemental experimental procedures

Document S2. Article plus supplemental information
