# Supplementary material for: Characterization of the epidermal-dermal junction in hiPSC-derived skin organoids
Source: Stem Cell Reports. 2022 May 12;17(6):1279–88. doi: 10.1016/j.stemcr.2022.04.008 (PMC9213820; doi:10.1016/j.stemcr.2022.04.008)
Supplement: Document S1. Figures S1–S4, Tables S1–S3, and Supplemental experimental procedures [file mmc1.pdf]

Stem Cell Reports, Volume *17*

Supplemental Information

Characterization of the epidermal-dermal junction in hiPSC-derived skin organoids

Veronika Ramovs, Hans Janssen, Ignacia Fuentes, Amandine Pitaval, Walid Rachidi, Susana M. Chuva de Sousa Lopes, Christian Freund, Xavier Gidrol, Christine L. Mummery, and Karine Raymond

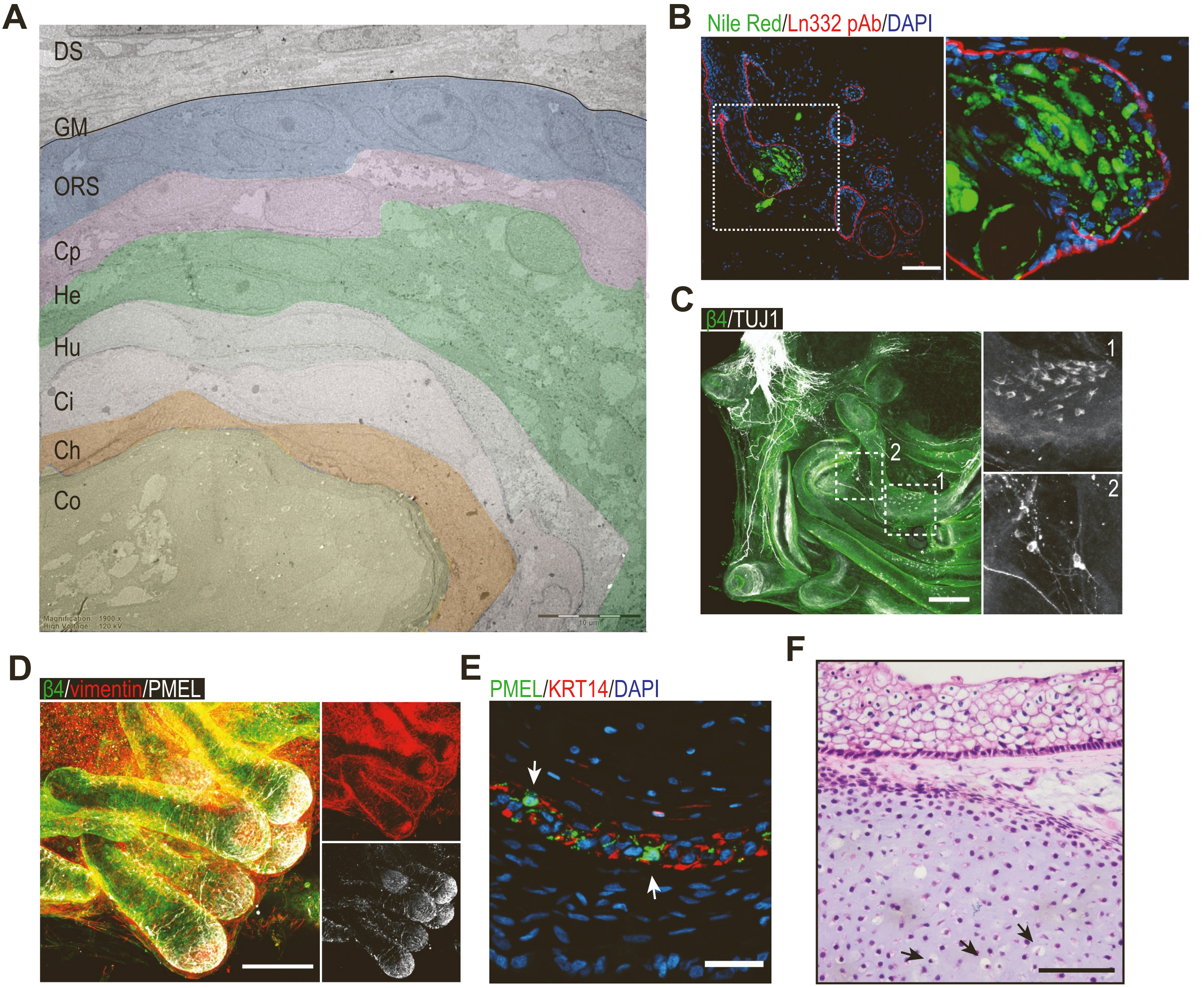

Figure S1: related to Figure 1

(**A**) TEM image of cross-section through a HF in skin organoid showing the following HF-specific layers: dermal sheath (DS), glassy membrane (GM), outer root sheath (ORS), companion layer (Cp), Henle's layer (He), Huxley's layer (Hu), inner root sheath cuticle (Ci) and cuticle (Ch). (**B**) Confocal image of section through skin organoid, stained with Nile Red, showing lipid-rich sebum localizing at sebaceous glands. Scale bar: 100μm. (**C**) Confocal images of organoid whole-mount stained with anti-β4 integrin subunit and anti-TUJ1 antibodies showing TUJ1+ neurons innervating HFs. Box 1 and 2 illustrate the presence of neuron soma and Merkel-like cells, respectively. Scale bars: 200μm. (**D**) Confocal images of organoid whole-mount stained with anti-β4 integrin subunit, anti-vimentin and anti-PMEL antibodies showing Vimentin+ fibroblasts and PMEL+ melanocytes in the HFs. Scale bar: 200μm. (**E**) Confocal image of section through skin organoid, showing PMEL+ melanocytes (arrows) in interfollicular epidermis. Scale bar: 30μm. (**F**) Representative bright field image of H&E-stained section of skin organoid showing hyaline cartilage. Arrows point to chondrocytes embedded in matrix within lacuna. (**A-F**) All organoids are imaged at day 130.

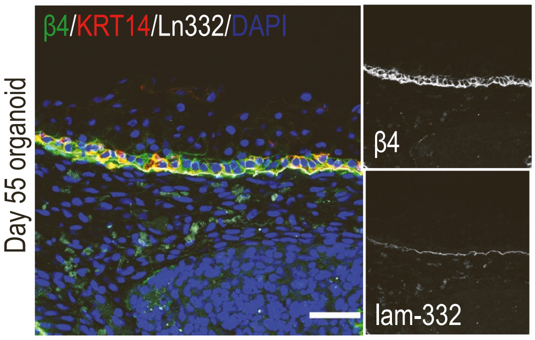

Figure S2: related to Figure 2
Confocal image of Day 55 organoid stained with anti β4 integrin subunit, anti-KRT14 and anti-Laminin-332 antibodies. Note that the laminin-332 localizes to the BM whereas integrin α6β4 can be found over the whole membrane of basal keratinocytes. Scale bar: 40μm.

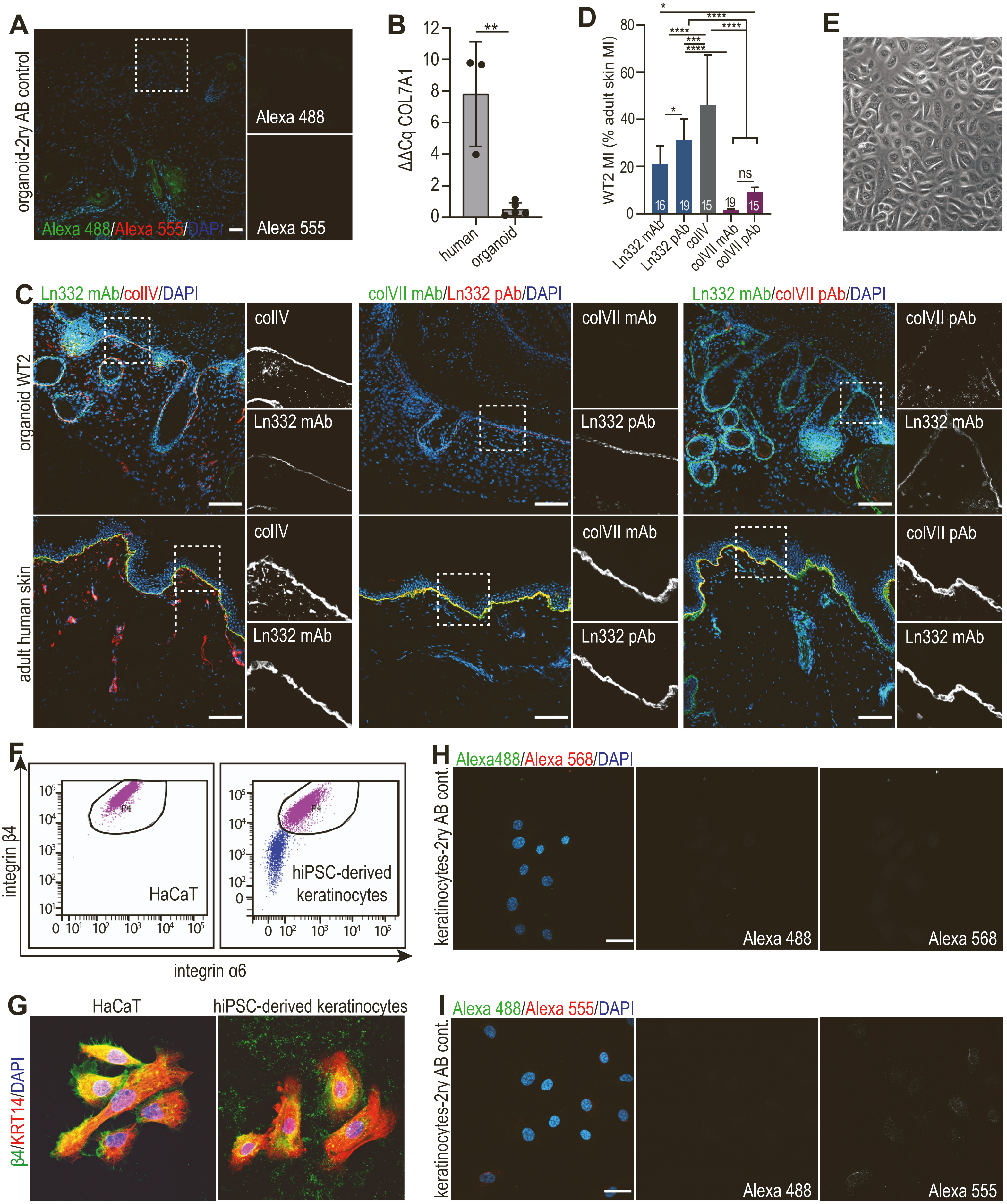
A
organoid-2ry AB control
Alexa 488/Alexa 555/DAPI
Alexa 488
Alexa 555
B
ΔΔCq COL7A1
**
human
organoid
D
WT2 MI (% adult skin MI)
Ln332 mAb
Ln332 pAb
colIV
colVII mAb
colVII pAb
ns
E
C
Ln332 mAb/colIV/DAPI
colVII mAb/Ln332 pAb/DAPI
Ln332 mAb/colVII pAb/DAPI
organoid WT2
adult human skin
colIV
Ln332 mAb
colVII mAb
Ln332 pAb
colVII pAb
F
integrin β4
integrin α6
HaCaT
hiPSC-derived keratinocytes
P4
G
HaCaT
hiPSC-derived keratinocytes
β4/KRT14/DAPI
H
Alexa488/Alexa 568/DAPI
keratinocytes-2ry AB cont.
Alexa 488
Alexa 568
I
Alexa 488/Alexa 555/DAPI
keratinocytes-2ry AB cont.
Alexa 488
Alexa 555

Figure S3: related to Figure 3

(**A**) Confocal images of Day 130 organoid stained with secondary antibodies only (2ry AB) showing the background control for images found in Fig. 3a, except when specified. Dashed box is amplified to the right. Scale bar: 40µm. (**B**) Normalized (ΔΔCq) mRNA expression of *COL7A1* is significantly decreased in organoids compared to adult human skin. mRNA expression of *LAMA3, LAMC2, COL4A1* and *COL4A2* was used for normalization. Each dot represents an organoid/skin sample and is an average of technical duplicate (mean ± SD, unpaired t test, P=0.0021). (**C**) Representative confocal images of organoid derived from WT2 hiPSCs (day130) and human adult skin sections stained with anti-laminin-332, anti-colIV and anti-ColVII antibodies. The expression of colVII is low and often non-uniform in the WT2 skin organoids, which is similar to the organoids derived from LUMCi045-A1 and LUMCi046-A1 hiPSC lines. Confocal images are presented as maximum projected z-stacks.
(**D**) Quantification of the laminin-332, colIV and colVII mean intensity (MI) in skin organoids (day 130) derived from WT2 line as compared to adult human skin. A total of 5-6 images of 3 independent differentiations of hiPSC or of human samples was used for quantifications (Tukey's multiple comparison, n = 15-19, *P<0.05, ***P<0.001, ****P<0.0001, mean ± SD). Scale bar: 100µm. (**E**) Bright field image of hiPSC derived keratinocytes. (**F**) Dot plots showing the population of integrin α6 and β4-positive hiPSC-derived keratinocytes that were sorted using flow cytometry. HaCaT keratinocytes were used as positive control. (**G**) Confocal images of FACS sorted hiPSC-derived keratinocytes stained for integrin β4 and keratin 14 (KRT14). HaCaT keratinocytes were used as positive controls. Scale bars: 30µm. (**H-I**) Confocal images of keratinocytes stained with secondary antibodies only (2ry AB) showing the backgound control for images found in fig. 3c: β4-Alexa 488 and ColVII mAb-Alexa 568 (**H**) and β4-Alexa 488 and ColVII pAb-Alexa 555 (**I**). Scale bars: 30µm.

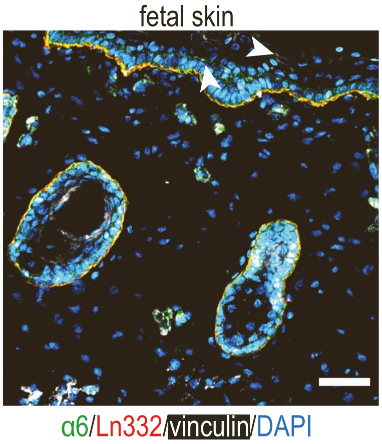

Figure S4: related to Figure 4
Confocal image of mid-gestational human skin, stained for vinculin, integrin α6 and laminin-332. Vinculin can be observed at basal membrane as well as in cell-cell contact of basal and suprabasal keratinocytes. Scale bar: 40μm.

EDJ component	Comparability of skin organoids to adult human skin
Integrins (α3β1 and α6β4)	✓
Type I HDs (plectin, BP230, α6β4; TEM)	✓
Vinculin	Localization: similar to mid-gestational period (cell-cell contacts, suprabasal)
Type IV collagen	✓ lower expression (40-80% of adult human skin)
Laminin-332	✓ lower expression (30-65% of adult human skin)
Type VII collagen	↓ non-homogeneous and very low expression (0.5-9% of adult human skin)
cell-cell adhesion	
Adherens junctions (E-cadherin)	✓
Desmosomes (Dsg-3; TEM)	✓
Tight junctions (ZO-1)	✓
Gap junctions (Cx43)	✓
Skin stratifications and morphology	
KRT5/KRT14	✓
KRT1/KRT10	✓
loricrin	✓
Rete ridges	✗

Table S1: related to Results & Discussion. Summary of how EDJ, cell-cell adhesion and skin stratification of skin organoids generated from lines LUMCi045-A1 and LUMCi046-A1 as compared to the adult human skin.

Antigen	Name	Type	Application	Dilution	Source
BP230	5E	Human mAb	IF	1:40	Kind gift of T. Hashimoto (Hashimoto et al., 1993)
CD90	55593	Mouse mAb	IF	1:100	BD Biosciences
Collagen type IV	Ab769	Goat pAb	IF	1:200	Millipore
Collagen type VII	LH7.7	Mouse mAb	IF	1:200	Sigma
Collagen type VII	Ab93350	Rabbit pAb	IF	1:100	Abcam
Connexin 43	ab11370	Rabbit pAb	IF	1:200	Abcam
Desmoglein-3	5G11	Mouse mAb	IF	1:100	Millipore
E-cadherin	H-108	Rabbit pAb	IF	1:100	Santa Cruz
FAP	AF3715	Sheep pAb	IF	1:50	R&D Systems
Itga3	J143	Mouse mAb	IF	1:200	(Fradet et al., 1984)
Itga6	GoH3	Rat mAb	IF	1:200	Kind gift of A. Sonnenberg (Sonnenberg et al., 1987)
Itga6-FITC	GoH3	RatmAb	FACS	1:50	BioLegend
Itgb1	TS2/16	Mouse mAb	IF	1:100	Santa Cruz
Itgb4	439-9B	Rat mAb	IF	1:200	BD Biosciences
Itgb4-PE	555720	Rat mAb	FACS	1:100	BD Biosciences
Keratin 1	HPA017917	Rabbit pAb	IF	1:250	Atlas Antibodies
Keratin 5	Ab53121	Rabbit pAb	IF	1:200	Abcam
Keratin 10	Poly19054	Rabbit pAb	IF	1:250	BioLegend
Keratin 14	PRB-155P	Rabbit pAb	IF	1:1000	BioLegend
Keratin 15	GP-CK15	Guinea pig pAb	IF	1:200	Progen
Laminin-332	R14	Rabbit pAb	IF	1:400	Kind gift of M. Aumailey
Laminin-332	P3H9-2	Mouse mAb	IF	1:200	Abcam
Loricrin	PRB-145P	Rabbit pAb	IF	1:250	BioLegend
Plectin	P1	Guinea pig pAb	IF	1:400	Kind gift of H. Herrmann (Stegh et al., 2000)
PMEL	NBP2-29407	Mouse mAb	IF	1:100	Novus
TUJ1	MMS-435P	Mouse mAb	IF	1:4000	Covance
Vimentin	D21H3	Rabbit mAb	IF	1:100	Cell SIgnaling
Vinculin	VIIF9	Mouse mAb	IF	1:5	Kind gift of M. Glukhova
ZO-1	61-7300	Rabbit pAb	IF	1:100	Invitrogen

Table S2: related to Experimental procedures. List of primary antibodies used, including application, dilution and source

Gene	Sequence primer forward	Sequence primer reverse	Source
COL7A1	5'-GTGAGGACTGCCCCTGAG-3'	5'- GACTCCACCTTCGAGACCC-3	(Murauer et al., 2011)
LAMA3	5'-ACTGGACTCACCTACGCCAACCCG-3'	5'-CCCTGTGAACTGGTTGCACTGTGG-3'	(Marionnet et al., 2006)
LAMC2	5'-GCCTTTTGGCACCTGTATTC-3'	5'-CAGGATTCTCATCCCCTGAA-3'	(Drake et al., 2010)
COL4A1	5'-GGATCGGCTACTCTTTTGTGATG-3'	5'-AAGCGTTTGCGTAGTAATTGCA-3'	(Wang et al., 2006)
COL4A2	5'-TGGACCTGATGGAAAGCGAG-3'	5'-CATTCCCCAGCGTCACCTTT-3'	Designed for this study

Table S3: **related to Experimental procedures.** List of primers used for RT-qPCR

SUPPLEMENTAL EXPERIMENTAL PROCEDURES

Differentiation of primary keratinocytes

Keratinocytes were differentiated from LUMCi045-A1 and LUMCi046-A1 as described previously (Guo et al., 2013). Briefly, clumps of hiPSC were seeded in StemFlex^{TM} medium on Matrigel-coated 6-well plates and incubated with defined keratinocyte serum-free medium (KSFM; ThermoFisher #10744019) containing 1µM all-trans Retinoic Acid (Sigma #R2625) and 10 ng/µl BMP4 (R&D #314-BP) for 4 days. Cultures were subsequently maintained in KSFM till day 13 when they were switched to Epithelial Proliferation Medium CnT-07 (Bio-Connect). Thirty days after seeding, cells were sorted for integrin $\alpha6^+$ integrin $\beta4^+$ population using fluorescence activated cell sorting (Becton Dickinson FACSAria cell sorter) to obtain a pure hiPSC-derived keratinocyte population that was maintained in CnT-07.

Antibodies

Primary antibodies used are listed in Table S2 and the secondary antibodies were: donkey anti-mouse Alexa 488, Donkey ati-mouse Alexa 568, donkey anti-mouse Alexa 647, donkey anti-rabbit Alexa 405, donkey anti-rabbit Alexa 488, donkey anti-rabbit Alexa 555, donkey anti-rabbit Alexa 647, donkey anti-rat Alexa 488, donkey anti-rat Alexa 555, donkey anti-guinea pig Alexa 488, donkey anti-goat Alexa 488 and donkey anti-human Alexa 647 (Invitrogen; dilution 1:250).

Quantification of the extracellular matrix components

For quantification of the extracellular matrix components, z-stacks with step size of 1.5µm (cryosections) or 1µm (keratinocytes) were acquired using GaAsP-PMT detector. The linear detection of fluorescent intensities of the detector was confirmed by manually measuring laser-power while changing the laser power settings. During the acquisition, settings were defined to be just below saturation intensity in the human skin samples. Region of interest (ROI) was delineated based on the laminin-332 staining of EDJ (cryosections) or phalloidin staining (keratinocytes) and the mean intensity of the ROI was calculated on the maximum intensity projection. To compare the expression levels in the organoids/fetal skin to those of human adult skin, the mean intensity (MI) of each organoid/fetal skin image (5-6 images/sample in total) was divided by the average MI of the adult samples (2-3 independent samples, 5-6 images/sample). To compare the expression levels in the hiPSC-derived-with primary keratinocytes, MI of 142-223 cells from 3-4 independent differentiation rounds was divided by the average MI of the primary keratinocytes (170 cells, 2 independent experiments).

RT-qPCR

RNA was extracted from human skin and organoids using NuceloSpin RNA kit (MACHEREY-NAGEL) according to the manufacturer's recommendations. Organoids were homogenized in lysis buffer using Pellet pestle Eppendorf and human skin using Polytron tissue homogenizer, at 4°C. 750 ng of purified RNA was used to synthesize the first-strand cDNA using iScript™ cDNA Synthesis Kit (BioRad #1708891). Quantitative PCR were performed using GoTaq® qPCR Master Mix (Promega #A600A) and C1000 Touch Thermal Cycler CFX384 Real Time PCR-system (BioRad). Analysis of the results was performed with CFX Manager software v 3.1 (BioRad). Results were presented as normalized expression (ΔΔCq, relative to zero), with *LAMA3*, *LAMC2*, *COL4A1* and *COL4A2* used as reference genes. Primers are listed in the Table S3.

SUPPLEMENTAL REFERENCES

Drake, J.M., Barnes, J.M., Madsen, J.M., Domann, F.E., Stipp, C.S., and Henry, M.D. (2010). ZEB1 Coordinately Regulates Laminin-332 and β4 Integrin Expression Altering the Invasive Phenotype of Prostate Cancer Cells *. Journal of Biological Chemistry *285*, 33940–33948.

Fradet, Y., Cordon-Cardo, C., Thomson, T., Daly, M.E., Whitmore, W.F., Lloyd, K.O., Melamed, M.R., and Old, L.J. (1984). Cell surface antigens of human bladder cancer defined by mouse monoclonal antibodies. Proc. Natl. Acad. Sci. U.S.A. 81, 224–228.

Guo, Z., Higgins, C.A., Gillette, B.M., Itoh, M., Umegaki, N., Gledhill, K., Sia, S.K., and Christiano, A.M. (2013). Building a microphysiological skin model from induced pluripotent stem cells. Stem Cell Res Ther 4 Suppl 1, S2.

Hashimoto, T., Amagai, M., Ebihara, T., Gamou, S., Shimizu, N., Tsubata, T., Hasegawa, A., Miki, K., and Nishikawa, T. (1993). Further analyses of epitopes for human monoclonal anti-basement membrane zone antibodies produced by stable human hybridoma cell lines constructed with Epstein-Barr virus transformants. J Invest Dermatol 100, 310–315.

Marionnet, C., Pierrard, C., Vioux-Chagnoleau, C., Sok, J., Asselineau, D., and Bernerd, F. (2006). Interactions between fibroblasts and keratinocytes in morphogenesis of dermal epidermal junction in a model of reconstructed skin. J Invest Dermatol *126*, 971–979.

Murauer, E.M., Gache, Y., Gratz, I.K., Klausegger, A., Muss, W., Gruber, C., Meneguzzi, G., Hintner, H., and Bauer, J.W. (2011). Functional Correction of Type VII Collagen Expression in Dystrophic Epidermolysis Bullosa. Journal of Investigative Dermatology *131*, 74–83.

Sonnenberg, A., Janssen, H., Hogervorst, F., Calafat, J., and Hilgers, J. (1987). A complex of platelet glycoproteins Ic and IIa identified by a rat monoclonal antibody. J. Biol. Chem. 262, 10376–10383.

Stegh, A.H., Herrmann, H., Lampel, S., Weisenberger, D., Andrä, K., Seper, M., Wiche, G., Krammer, P.H., and Peter, M.E. (2000). Identification of the Cytolinker Plectin as a Major Early In Vivo Substrate for Caspase 8 during CD95- and Tumor Necrosis Factor Receptor-Mediated Apoptosis. Mol Cell Biol 20, 5665–5679.

Wang, T.-W., Sun, J.-S., Huang, Y.-C., Wu, H.-C., Chen, L.-T., and Lin, F.-H. (2006). Skin basement membrane and extracellular matrix proteins characterization and quantification by real time RT-PCR. Biomaterials *27*, 5059–5068.
